# Platform-dependent effects of genetic variants on plasma APOL1 and their implications for kidney disease

**DOI:** 10.1101/2025.01.30.635763

**Published:** 2025-02-02

**Authors:** Qingbo S. Wang, Jinguo Huang, Leanne Chan, Nicole Haste, Niclas Olsson, Aleksandr Gaun, Fiona McAllister, Deepthi Madhireddy, Amos Baruch, Eugene Melamud, Anastasia Baryshnikova

## Abstract

Mutations in apolipoprotein L1 (APOL1) are strongly associated with an increased risk of kidney disease in individuals of African ancestry, yet the underlying mechanisms remain largely unknown. Plasma proteomics provides opportunities to elucidate mechanisms of disease by studying the effects of disease-associated variants on circulating protein levels. Here, we examine the genetic drivers of circulating APOL1 in individuals of African and European ancestry from four independent cohorts (UK Biobank, AASK, deCODE and Health ABC) employing three proteomic technologies (Olink, SomaLogic and mass spectrometry). We find that disease-associated *APOL1* G1 and G2 variants are strong pQTLs for plasma APOL1 in Olink and SomaLogic, but the direction of their effects depends on the proteomic platform. We identify an additional *APOL1* missense variant (rs2239785), common in Europeans, exhibiting the same platform-dependent directional discrepancy. Similarly, variants in the kallikrein-kinin pathway ( *KLKB1* , *F12* , *KNG1* ) and their genetic interactions exhibit strong *trans* -pQTL effects for APOL1 measured by Olink, but not SomaLogic. To explain these discrepancies, we propose a model in which *APOL1* mutations and the kallikrein-kinin pathway influence the relative abundance of two distinct APOL1 forms, corresponding to APOL1 bound to trypanolytic factors 1 and 2, which are differentially recognized by different proteomic platforms. We hypothesize that this shift in relative abundance of APOL1 forms may contribute to the development of kidney disease.

## Full Text Availability

The license terms selected by the author(s) for this preprint version do not permit archiving in PMC. The full text is available from the preprint server.

